# Start spreading the news: a deliberate approach to POCUS program development and implementation

**DOI:** 10.1186/s13089-023-00309-6

**Published:** 2023-03-09

**Authors:** Mathilde Gaudreau-Simard, Elaine Kilabuk, Samantha Halman, Krista Wooller, Michael Y. Woo, Robert Arntfield, Irene Ma, Alan J. Forster

**Affiliations:** 1 Division of General Internal Medicine, Department of Medicine, The Ottawa Civic Hospital, University of Ottawa, 1053 Carling Ave, D 107, Box 209, Ottawa, ON K1Y 4E9 Canada; 2 The Ottawa Hospital Research Institute, The Ottawa Hospital, 725 Parkdale Ave, Ottawa, ON K1y 4E9 Canada; 3 Division of General Internal Medicine, Department of Medicine, The Ottawa General Hospital, University of Ottawa, 501 Smyth Road, Ottawa, ON K1Y 4E9 Canada; 4 Department of Emergency Medicine, The Ottawa Civic Hospital, University of Ottawa, 1053 Carling Ave, Ottawa, ON K1Y 4E9 Canada; 5 Division of Critical Care, London Health Sciences Center, Victoria Hospital, Western University, 800 Commissioners Road East, Room D2 521A, London, ON N6A 5W9 Canada; 6 Division of General Internal Medicine, Department of Medicine, University of Calgary, 3330 Hospital Dr NW, Calgary, AB T2N 4N1 Canada; 7 Department of Community Health Sciences, University of Calgary, 3330 Hospital Dr NW, Calgary, AB T2N 4N1 Canada

## Abstract

While there is an expanding body of literature on Point-of-Care Ultrasound (POCUS) pedagogy, administrative elements that are necessary for the widespread adoption of POCUS in the clinical environment have received little attention. In this short communication, we seek to address this gap by sharing our institutional experience with POCUS program development and implementation. The five pillars of our program, selected to tackle local barriers to POCUS uptake, are education, workflow, patient safety, research, and sustainability. Our program logic model outlines the inputs, activities, and outputs of our program. Finally, key indicators for the monitoring of program implementation efforts are presented. Though designed for our local context, this approach may readily be adapted toward other clinical environments. We encourage others leading the integration of POCUS at their centers to adopt this approach not only to achieve sustainable change but also to ensure that quality safeguards are in place.

## Main text

Though Point-of-Care Ultrasound (POCUS) program leaders are generally well versed in POCUS education, they may lack the tools to support the broader implementation of POCUS in the clinical environment. While there is an expanding body of literature on POCUS pedagogy [1–3], administrative and logistical elements that are necessary for the widespread adoption of POCUS have received little attention [4, 5]. Considering the value proposition of POCUS to enhance patient care [6], we set out to increase the uptake of POCUS by general internists at the Ottawa Hospital, a tertiary care academic center. Using concepts from the literature on change management, quality improvement, and program evaluation, we developed a comprehensive approach to program development and implementation. In this paper, we share our approach as a model to support others looking to achieve the safe uptake of POCUS at their institution.

### Understand your local environment

The first step to any change initiative is to gain an understanding of the operational environment [7]. A thorough understanding of local barriers and enablers, including stakeholder perceptions and readiness for change [8–10], organizational culture, and infrastructure is crucial [7].

Our program stakeholders include senior management, divisional leadership, content experts, non-clinical partners (biomedical engineering and information technology services), and end users. Stakeholder engagement was achieved using different mediums including informal interviews, divisional meetings, and online surveys.

The Ottawa Hospital has established programs in Emergency Medicine Ultrasonography (EMUS) and Critical Care Ultrasonography (CCUS). In addition to offering a wealth of experience in program development, these programs have a mature POCUS infrastructure, including hospital-based archiving, that can readily be expanded to other departments. Our environmental survey also showed that there is strong leadership support both at the senior management and divisional level for the implementation of POCUS in General Internal Medicine (GIM).

In addition to these enablers, we identified barriers to the broader uptake of POCUS in our division. Similar to barriers that have previously been described [11], lack of training, lack of time, lack of quality safeguards, and lack of evidence were quoted as being prohibitive. Finally, we identified that previous attempts to integrate POCUS in the division had been unsuccessful due to the lack of sustained efforts.

### Develop and communicate a vision of change

Once we had developed a good understanding of our local barriers and enablers, we set out to establish our mission, values, and vision (Table 1) [12]. These are aligned with our organization’s strategic goals [13] and will give direction to our change efforts [14, 15].

**Table 1:** Mission, values, and vision

Mission	Leverage POCUS to provide better value care, enhance patient and provider experience, and achieve better health of populations
Values	Quality and patient safety Educational excellence Sustainability
Vision	To achieve widespread safe use of POCUS by general internists at the Ottawa Hospital

### Remove obstacles [14]

Our next step was to identify strategies that would address each barrier (Table 2). This exercise allowed us to come up with the five overarching pillars of our program.

**Table 2:** GIM POCUS program goals and pillars

Barrier	Goals	Program pillars
Lack of training	Deliver educational activities to allow internists to gain the cognitive and psychomotor competencies required to perform and integrate POCUS clinically	Education
Lack of time	Establish a seamless POCUS workflow that is adapted to the high clinical volumes faced by internists	Workflow
Lack of quality safeguards	Establish a quality and patient safety program	Patient safety
Lack of evidence	Generate local data on clinical effectiveness, efficiency, and relevance of POCUS	Research
Lack of sustained efforts	Build capacity within the division and foster strong interdepartmental collaboration	Sustainability

### Plan program resources, activities, and outputs

Once we had identified the key elements of our program, we set out to plan our specific deliverables [16]. We present a logic model for our program (Table 3). A logic model is a systematic and visual way to outline the different elements of a program, from the inputs required to operate the program, the activities the program will deliver, and the outputs that will result from program implementation [17].

**Table 3:** GIM-POCUS program logic model: resources, activities, and outputs

Resources/Inputs	Activities	Outputs	Pillars
**Early adopters**^1^ **Infrastructure**: 1. Ultrasound machines on wheels on medical wards 2. Hand held devices that allow for portability 3. Archiving capabilities **Funding**: 1. POCUS leads -Protected teaching, administrative and research time 2. Non-clinical partners -Biomedical engineering department -Information system department 3. Academic grants to support research, quality improvement and innovation	POCUS academic half days	Establish a POCUS curriculum imbedded within the GIM residency training program	Education
	POCUS rotation		
	Asynchronous feedback on archived scans		
	GIM POCUS rounds	Continuous professional development for practicing attendings	
	POCUS course (interdepartmental)		
	Bedside scanning sessions		
	Optimize the physical location of US machines	Optimize the physical environment	Workflow
	Optimize the US to user ratio		
	Integrate an archiving platform with US machines and the hospital information system	Onboard users to an archiving platform	
	Training sessions on the use of the archiving platform		
	Establish a system failure reporting process for US machines and the archiving platform	Maintenance of infrastructure	
	Establish standards for what constitutes an adequate scan^2^	Quality assurance of scans	Patient safety
	Establish and implement a credentialing process^3^		
	Establish a mechanism by which a proportion of scans performed by credentialed users are reviewed		
	Adopt patient safety policies^4^	POCUS quality improvement program	
	Conduct morbidity and mortality rounds for POCUS-related adverse events		
	Implement an adverse event reporting and reviewing process		
	Develop questions and set up projects that are specific to the use of POCUS in GIM	Research program with a focus on quality improvement and implementation science	Research
	Develop an IM POCUS fellowship	Capacity building	Sustainability
	Recruit and retain credentialed users		
	Interdepartmental rounds	Cross-departmental collaboration	
	Interdepartmental delivery of teaching activities (course, academic half days)		

US = ultrasound

^1^Locally, the early adopter groups are POCUS-trained internists who have completed dedicated POCUS training (ranging from 3 to 6 months) as part of their GIM subspecialty residency training.

^2^Including standards for image acquisition, image interpretation, clinical integration, and documentation

^3^There is currently no standardized credentialing process for Internal Medicine POCUS in Canada. We, therefore, developed a dedicated POCUS Entrustrable Professional Activities (EPA) using consensus methodology. To be considered credentialed, learners must achieve entrustment on 50 EPAs, including a minimum attributed to each core application.

^4^Including learner policy, incidental findings policy, infection prevention policy

### Monitor

Finally, we planned for monitoring of our implementation efforts. We selected indicators that could feasibly be collected, would adequately signal change, and would be actionable (Table 4) [18–20].

**Table 4:** Indicators to monitor program implementation

Pillar	Indicator	Frequency
Education	# of credentialed GIM trainees	Annually
	# of credentialed GIM attendings	Annually
Workflow	# of archived scans by credentialed users	Quarterly
	# of system failures reported to IS and biomed	Quarterly
	Level of agreement with “Our POCUS infrastructure (machines and archiving) facilitates the safe use of POCUS in patient care”	Annually
Patient safety	% of scans performed by credentialed users that meet quality assurance standards	Quarterly
	# of reported POCUS-related adverse events	Quarterly
Research	# POCUS publications with GIM as principal investigator	Annually
	# POCUS grants with GIM as principal investigator	Annually
Sustainability	# of POCUS-fellowship trained internists in the division	Annually
	# of credentialed internists participating in the delivery of the training program	Annually
	# of non-internists participating in the delivery of the training program	Annually

IS = information services

## Conclusion

In this paper, we have—through sharing our institutional experience—sought to address a gap in the literature regarding POCUS implementation in the clinical environment. A strength of our program is its focus on quality and patient safety. Our program is designed specifically for our local context but may readily be adapted toward other clinical environments. As such, we encourage others leading the integration of POCUS at their centers to adopt this approach not only to achieve sustainable change but also to ensure that appropriate quality safeguards are in place.

## Data Availability

Data sharing is not applicable to this article as no datasets were generated or analyzed during the current study.
